# Impact of COVID-19 pandemic on psychiatric emergency care in a general hospital

**DOI:** 10.1192/j.eurpsy.2023.873

**Published:** 2023-07-19

**Authors:** J. M. Rodríguez Capilla, A. Rubio Carramiñana, S. Vega Castellote, S. López Fernández, I. Arilla Herrera, J. M. Almenara Galdeano, A. Mora Prat, M. Campillo Benito, J. Albero Garcia, A. Valderrey Ratia, A. Grau Peñas, C. Pastor Fernández, M. Moreno Monzó, J. Guitart Gil, J. Martínez Raga, C. Knecht

**Affiliations:** 1 Psiquiatría, Hospital Doctor Peset, Valencia, Spain

## Abstract

**Introduction:**

The SARS-CoV-2 pandemic has produced an unprecedented clinical situation, causing a direct and indirect impact on the physical and mental health of the population. In Spain, between March 15 and June 21 of 2020, it was decreed a home confinement that caused the interruption of the daily life of millions of people. However, there are few studies that analyze the changes produced in psychiatric care in the Emergency Department (ED).

**Objectives:**

To analyze the changes produced in psychiatric emergencies, subanalysing paediatric population, during the first year of the pandemic (COV1/Y-COV1) compared to the previous year (NOCOV/Y-NOCOV1). To analyze the clinical features of patients attended during the lockdown period of the pandemic (LOCK) and compare it to the period of the pandemic after the lockdown (NOLOCK).

**Methods:**

Through the registry of computerized medical records, patients who attended the psychiatric hospital emergency department between 03/01/2019 and 02/28/2021 were identified. We also identified all attendances from 15/03 to 21/06 in 2019 and in 2020 to obtain variables from the lockdown period.

**Results:**

During period of this study, 2694 psychiatric visits made in the ED (1744 patients - 54.3% women, and 69.5% were between 25 and 64 years-), 1537 in NOCOV and 1157 in COV1. Significant differences were found between COV1 and NOCOV in sociodemographic variables, such as employment status and number of offspring. At a clinical level, in COV1, we observed an increase in attendance due to heteroaggressive behaviors, mania, insomnia and problems due to substance use. An increase in the prescription and/or modification of treatment was observed (59.3% vs 54.3%). During COV1, in terms of discharge follow-up in the month following the ED visit, telematic assistance increased (11.4% vs. 5.3%). During the period of study, 282 ED attendances were performed, 153 in Y-NOCOV and 129 in Y-COV1. At a clinical level, during Y-COV1, a decrease in attendances related to substance use was found significant. The sub-analysis carried out for LOCK and NOLOCK yields similar data to those obtained in the COV1 vs. NOCOV1 comparison. During lockdown, the face-to-face follow-up in the month following the ED was significantly lower (39,5% vs 57,1%) regarding telematic follow-up (24,4% vs 5,8%) In this period, an increase of adolescents without previous mental health follow-up was observed (44% LOCK vs. 22% NOLOCK).

**Conclusions:**

Our work supports the hypothesis that the COVID-19 pandemic caused a change in psychiatric care in the ED. It also shows how lockdown changed the attendance in psychiatric emergencies, and also in the later community care attendance. Changes are detected in emergency care for adolescents during the pandemic compared to the previous year. Strikingly, our study does not reflect a quantitative increase in the demand. It would be of interest to continue collecting data after the time of the present project.

**Disclosure of Interest:**

None Declared

